# Analyzer-based X-ray phase contrast imaging using the forward-diffracted o-beam in a few-millimetre-thick Bragg-case asymmetrically cut crystal

**DOI:** 10.1107/S1600576725002985

**Published:** 2025-04-25

**Authors:** Marcelo Goncalves Honnicke, Juliana Manica, XianRong Huang

**Affiliations:** a Universidade Federal da Integracao Latino-Americana Foz do Iguacu Parana85867-970 Brazil; b Advanced Photon Source Argonne National Laboratory 9700 South Cass Avenue Argonne IL60439 USA; SLAC National Accelerator Laboratory, Menlo Park, USA

**Keywords:** enhanced anomalous transmission, analyzer-based X-ray phase contrast imaging, forward-diffracted o-beam, thick crystals

## Abstract

An analyzer-based X-ray phase contrast imaging experiment employing the forward-diffracted o-beam in a few-millimetre-thick Bragg-case asymmetrically cut analyzer crystal has been implemented and tested in a geometry similar to that used in conventional radiography. The high angular stability requirements were overcome using specially designed crystals and a closed-loop feedback intensity system, monitoring the intensity of the diffracted h-beam. Analyzer-based images (ABis) taken at different angular positions on the forward-diffracted o-beam rocking curve show different contrasts, as expected. However, the ABis did not show sharp borders. This was closely investigated by X-ray topography and Δ*d*/*d* mapping and was attributed to the stresses caused by the analyzer crystal’s own weight in the bulk. Further investigation of the crystal design using finite element analysis coupled with dynamical theory of X-ray diffraction is envisaged.

## Abstract 2

An analyzer-based X-ray phase contrast imaging experiment employing the forward-diffracted o-beam in a thick Bragg-case asymmetrically cut analyzer crystal has been implemented and tested in a geometry very similar to that used in conventional radiography.

## 1. Introduction

**Figure 1:**
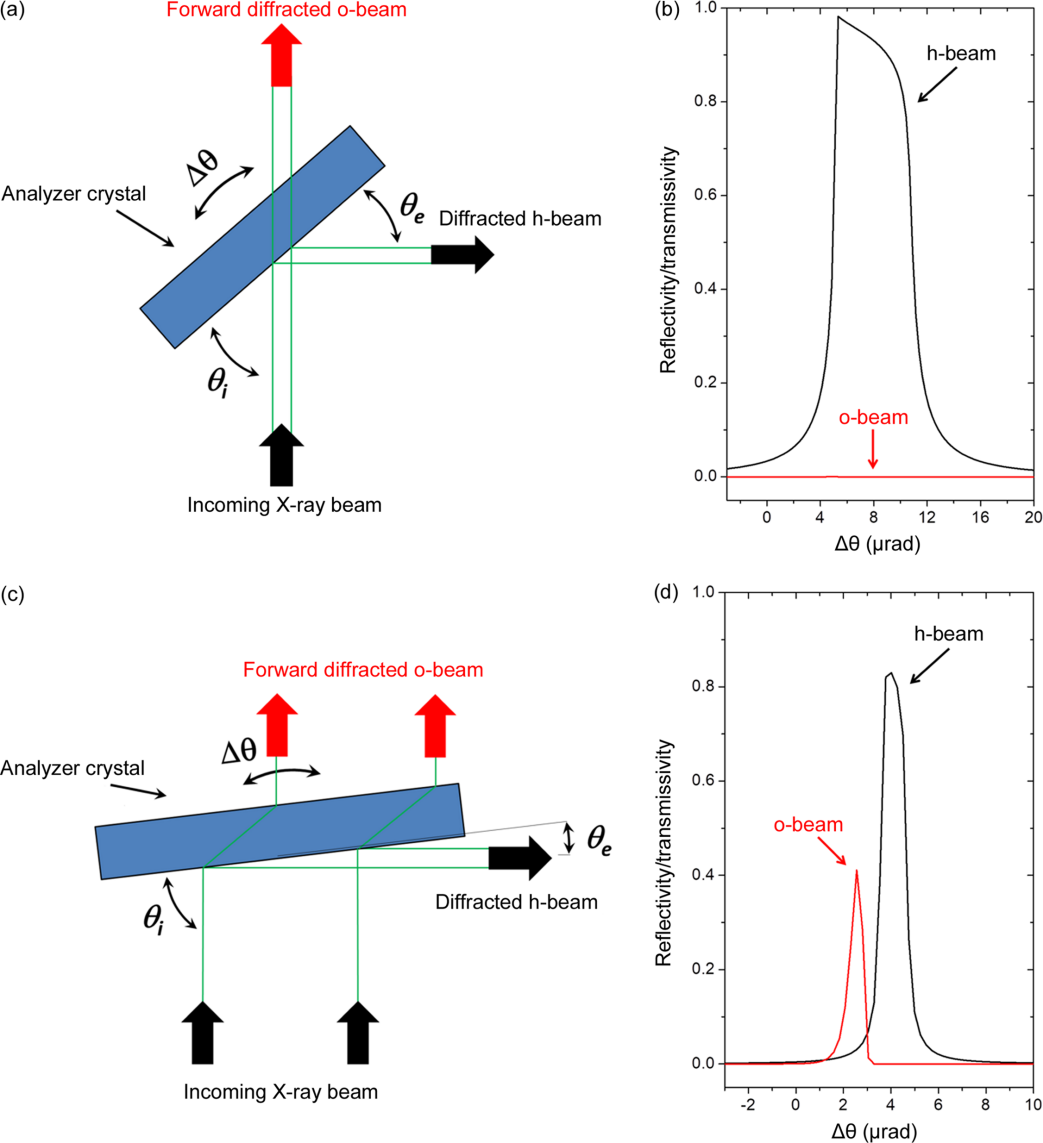
(*a*) Schematic of symmetric Bragg diffraction. (*b*) Example of diffracted h-beam and forward-diffracted o-beam profiles, calculated by the dynamical theory of X-ray diffraction for a 3 mm-thick Si symmetric single crystal with 444 reflection at 11.08 keV. (*c*) Schematic of asymmetric Bragg diffraction with a grazing emergence diffracted h-beam. (*d*) Example of diffracted h-beam and forward-diffracted o-beam profiles calculated by the extended dynamical theory of X-ray diffraction (Huang *et al.*, 2013 ▸) for a 3 mm-thick Si asymmetric single crystal with 444 reflection (*b* = −0.035) at 11.08 keV.

Anomalous transmission, detected in either Laue-case or Bragg-case diffraction, has been observed and studied for a long time (Borrmann, 1941 ▸; Borrmann, 1950 ▸; Borrmann, 1955 ▸; Wagner, 1956 ▸). The super-Borrmann effect, which is the enhanced anomalous transmission for a more than two-beam case (Lang, 1998 ▸; Authier, 2001 ▸), has also been observed and studied (Borrmann & Hartwig, 1965 ▸; Hildebrandt, 1967 ▸) and recently revisited for topography studies on Ge crystals (Matsui *et al.*, 2022 ▸). Enhanced anomalous transmission (EAT) of the forward-diffracted o-beam for asymmetrically cut crystals with the diffracted h-beam at grazing emergence has been reported also for the Laue case (Kishino *et al.*, 1972 ▸; Härtwig, 1976 ▸; Härtwig, 1977 ▸) and Bragg case (Kishino, 1971 ▸; Bedyńska, 1973 ▸; Härtwig, 1981 ▸). The strong sensitivity of EAT to lattice defects has also been reported (Kishino, 1974 ▸). EAT has been applied in ultra-high-resolution monochromators/analyzers for inelastic X-ray scattering (Shvyd’ko *et al.*, 2006 ▸; Cai *et al.*, 2013 ▸). Note that such an effect cannot be simulated on the basis of the simplified dynamical theory of X-ray diffraction (two-beam case); the extended dynamical theory of X-ray diffraction (Huang *et al.*, 2013 ▸) is required (Fig. 1 ▸).

Phase contrast X-ray imaging is a well established technique currently used in a variety of applications (Scopel *et al.*, 2015 ▸; Gobo *et al.*, 2024 ▸; Perez Vargas *et al.*, 2024 ▸). Among phase contrast X-ray imaging techniques (Ando & Hosoya, 1972 ▸; Förster *et al.*, 1980 ▸; Wilkins *et al.*, 1996 ▸; Pfeiffer *et al.*, 2006 ▸; Olivo & Speller, 2007 ▸), analyzer-based X-ray phase contrast imaging (ABI) (Förster *et al.*, 1980 ▸; Davis *et al.*, 1995 ▸) is one of the simplest techniques to implement, despite the high angular stability requirements, since no requirements on source coherence are demanded.

The use of thick asymmetrically cut crystals as the analyzer, with the diffracted h-beam at grazing emergence, brings advantages to the ABI setup. The analyzer-based images (ABis) are collected at different angular positions on the forward-diffracted o-beam rocking curve [Figs. 1 ▸(*c*) and 1 ▸(*d*)] in a geometry very similar to the conventional attenuation radiography setup, *i.e.* the detector surface is set after the sample and normal to the X-ray beam (the difference is the analyzer crystal which is set between the sample and detector). Since the analyzer crystal is thick (a few millimetres), the purely transmitted beam (attenuated mainly by the photoelectron absorption) is supressed. Another advantage is that the grazing emergence diffracted h-beam can be used as a closed-loop feedback intensity system to keep the analyzer crystal angular position fixed when acquiring images.

**Figure 2:**
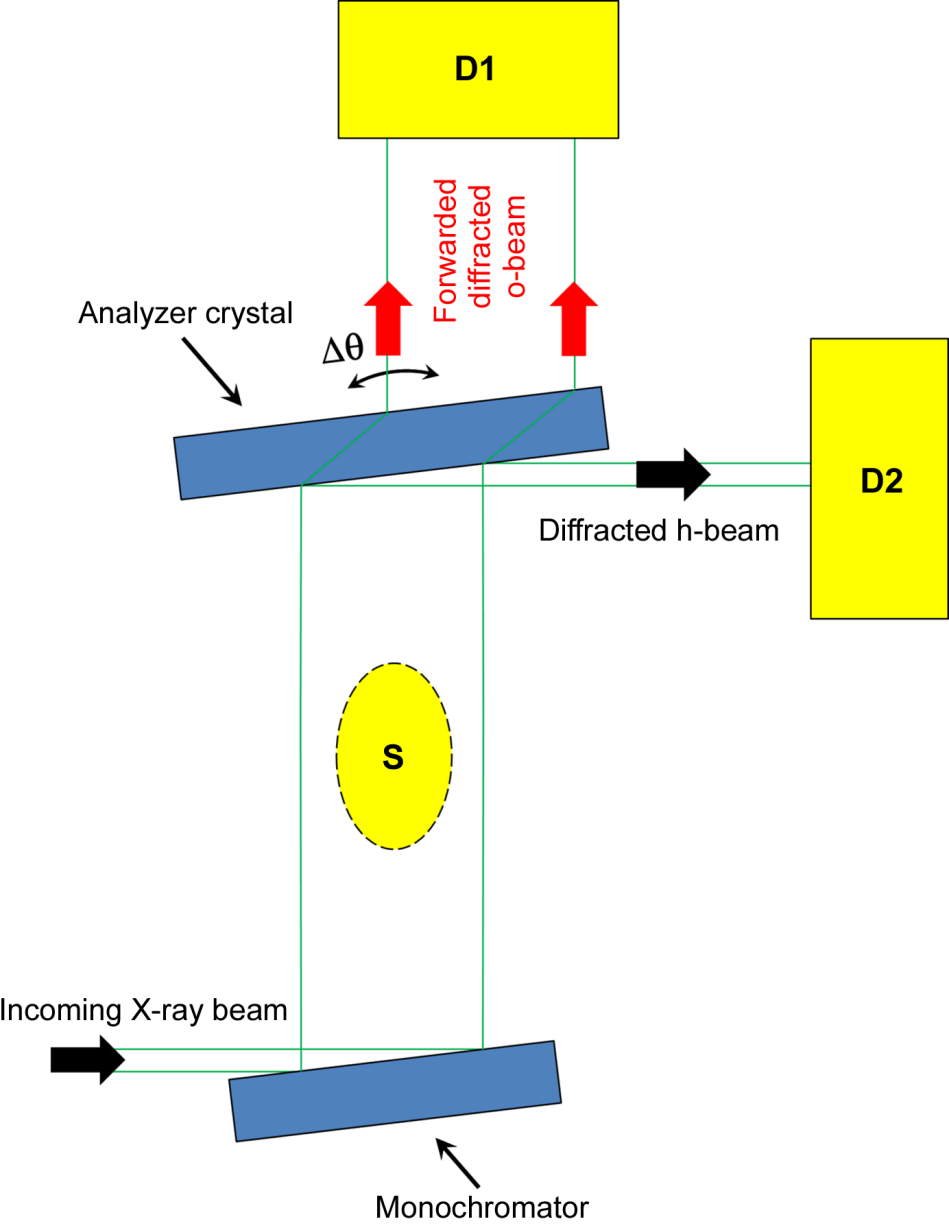
Schematic of the proposed experiments for mounting and testing the forward-diffracted o-beam ABI setup. S – sample, D1 – detector 1, D2 – detector 2. For characterizing the forward-diffracted o-beam, D1 and D2 are scintillator detectors and S is excluded. For acquiring ABis with the forward-diffracted o-beam, S is included, D1 is an area detector (CCD) and D2 is a scintillator detector. For forward-diffracted o-beam double-crystal topography, D1 is a CCD, D2 is a scintillator detector and S is excluded.

Herein, we propose to use the EAT in a few-millimetre-thick Bragg-case asymmetrically cut analyzer crystal for mounting and testing a forward-diffracted o-beam ABI setup, as schematically shown in Fig. 2 ▸.

## 2. Crystal preparation and experiment for characterizing the forward-diffracted o-beam

**Figure 3:**
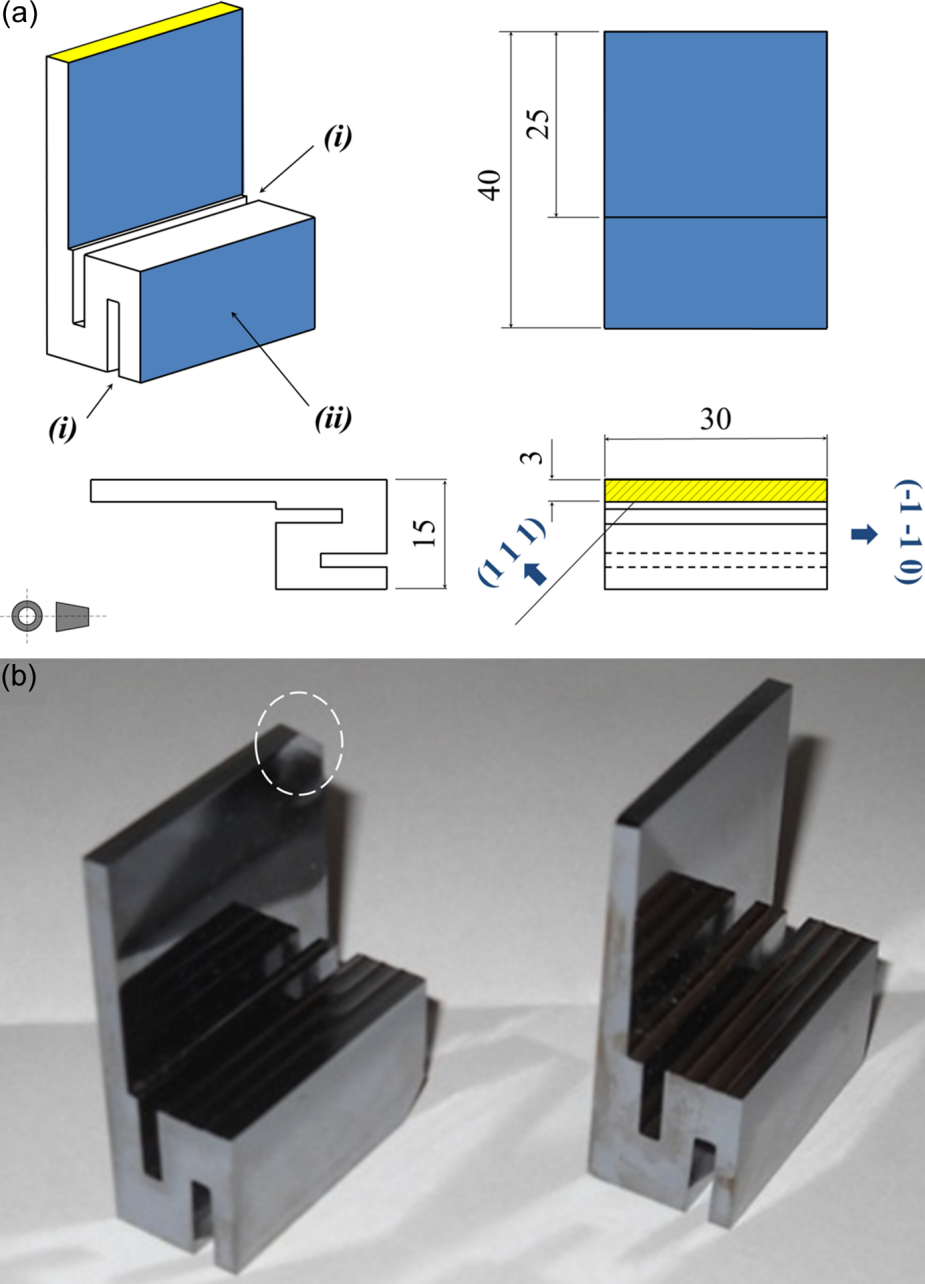
(*a*) Sketch of the crystals designed for the experiment showing (i) the strain reliefs and (ii) the surface used for fixture, in order to avoid any spurious stresses in the diffraction bulk. (*b*) Crystals ready (after cutting, lapping and etching) to be mounted in the double-axis diffractometer for the EAT experiments. There is a small chamfer (dashed circle) in the crystal on the left. This is due the Si ingot border that was reached when cutting the crystal, but does not affect the functionality of the crystal, since the imaged area was ∼25 × 25 mm^2^.

As mentioned previously, EAT is very sensitive to lattice defects (Kishino, 1974 ▸). Specially designed crystals are therefore needed in order to avoid stresses in the diffracted crystal volume (stresses due to the crystal fixture and the crystal’s own weight). Hence crystals with heavy bases and strain releases were designed as shown in Fig. 3 ▸. The fixture surface is kept far away from the diffraction volume. Both crystals were produced from an Si(111) 8 kΩ cm resistivity floating-zone ingot provided by Wacker (Siltronic). It was oriented and cut at −43.54° from the (111) surface, towards 3° off the [112] direction to avoid spurious diffraction effects (glitches) in the image background. In this way, at 11.08 keV the Si 444 diffraction occurs at 45.54° with the angle of incidence θ_i_ = 89.08° (Fig. 1 ▸) and the angle of emergence *θ*_e_ = 2° (asymmetry factor *b* = −28.6). The crystals were cut with resin bond diamond blades. Subsequently, they were lapped (SiC abrasive, grit 800) and then etched (HF:HNO_3_:CH_3_COOH, 1:20:1) at 298 K for 1 min a total of three times to remove the damaged layer due to cutting and lapping.

**Figure 4:**
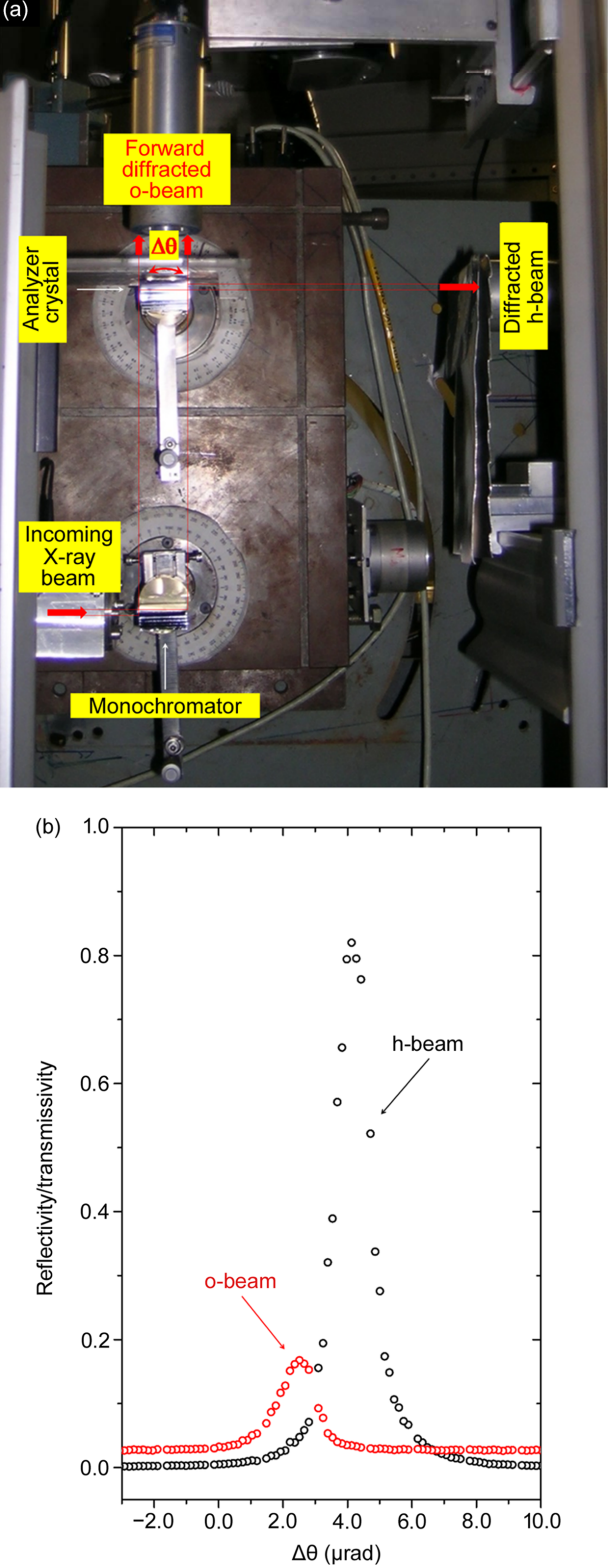
(*a*) 3 mm-thick Si asymmetric crystals with 444 reflection, mounted in the double-axis diffractometer for the EAT measurements. Both the forward-diffracted o-beam and the diffracted h-beam were measured by scintillation detectors. (*b*) Measured forward-diffracted o-beam (open red circles) and diffracted h-beam (open black circles) profiles.

The experiment setup (Fig. 4 ▸) was mounted at the XRD2 beamline at Laboratório Nacional de Luz Sincrotron (LNLS) (Giles *et al.*, 2003 ▸). A non-dispersive double-crystal setup, with a first asymmetrically cut Si crystal (444 reflection, *b* = −0.035) to expand the beam and a second asymmetrically cut Si crystal (444 reflection, *b* = −28.6) as a forward-diffracted o-beam Bragg-case analyzer crystal, was mounted on a double-axis diffractometer (Hart, 1980 ▸). This instrument has a precision of 0.3 µrad per step and thermomechanical stability better than 0.1 µrad h^−1^ (Hönnicke *et al.*, 2007 ▸). The incoming X-ray beam on the ABI setup has a divergence of 40 µrad in the vertical scattering plane and 750 µrad in the horizontal scattering plane for a beam size of 0.9 × 25 mm^2^ with a bandwidth Δλ/λ ≃ 1.10^−4^, delimited by the beamline double-crystal monochromator (Si 111). The Bragg-case analyzer crystal alignment was done by simultaneously measuring the diffracted h-beam and the forward-diffracted o-beam [Fig. 4 ▸(*b*)] with scintillation detectors (FMB Oxford). Note that a very similar experiment can be mounted with a conventional source using a slightly lower energy (Cu *K*α, 8.04 keV) and lower asymmetry factor (*b* = −20) with third-order diffraction (Si 333). The unique requirement for imaging applications is a proper collimation for a long fine-focus diffraction X-ray tube, working in point focus with a 1.10 m collimator with collimation slits of 0.3 (*H*) × 12 (*V*) mm^2^ (Hönnicke *et al.*, 2012 ▸) to avoid image distortions.

## 3. Analyzer-based X-ray phase contrast imaging with the forward-diffracted o-beam

**Figure 5:**
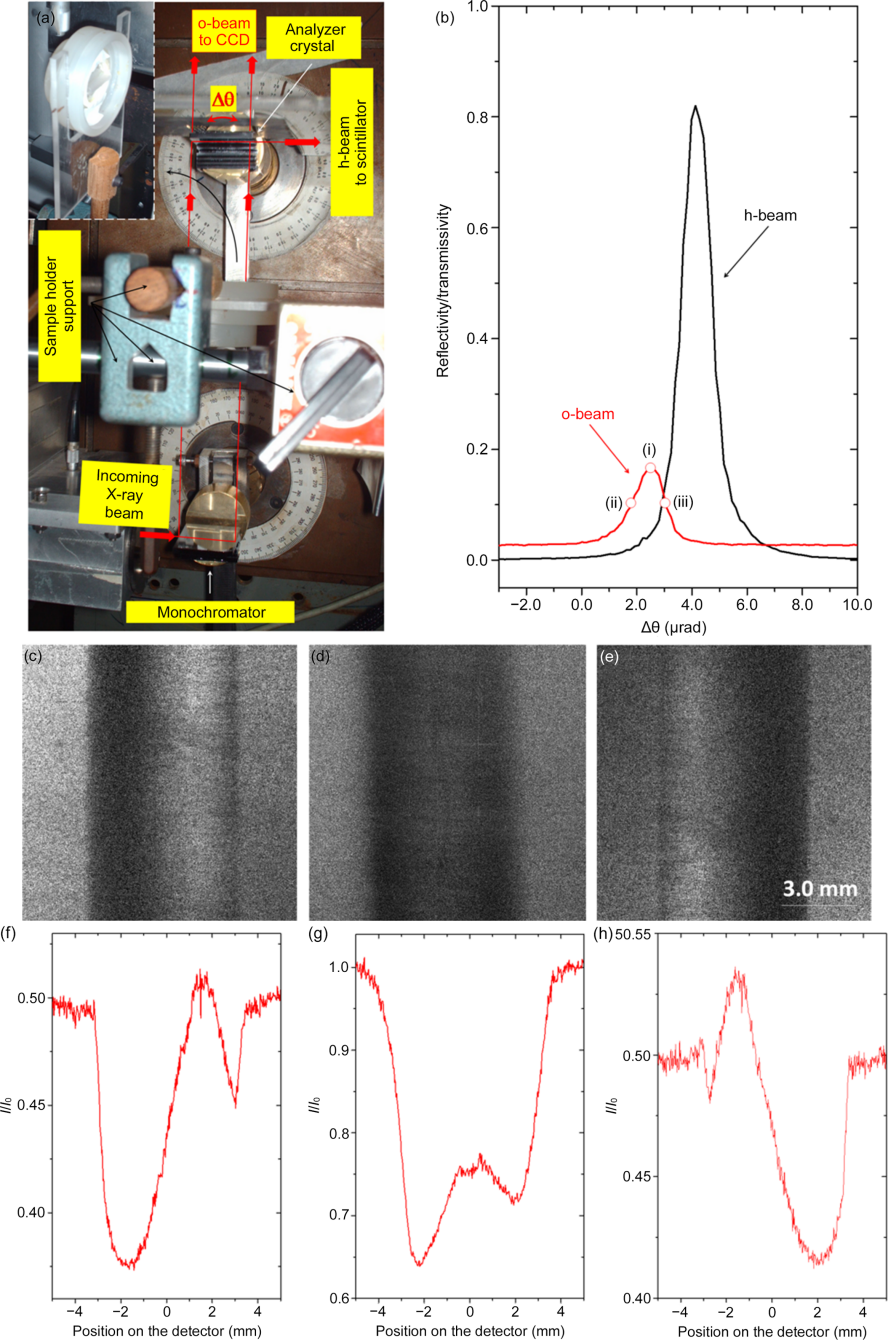
(*a*) Experimental ABI setup with the forward-diffracted o-beam, showing the sample holder in the inset (top view). (*b*) Diffracted h-beam and forward-diffracted o-beam showing the angular positions where the ABis of a polypropyl­ene tube (*c*)–(*e*) were acquired. (*f*)–(*h*) Cross sections of ABis (*c*)–(*e*).

The ABI setup with the forward-diffracted o-beam uses an imaging detector set in the forward-diffracted o-beam of the Bragg-case analyzer crystal (Fig. 5 ▸); however, a scintillation detector is kept in the grazing emergence diffracted h-beam for monitoring the intensity with a closed-loop feedback intensity system to keep the analyzer crystal angular position fixed when collecting the ABis with the forward-diffracted o-beam. The images were acquired using a direct conversion 1242 × 1152 pixel CCD detector (Princeton Instruments) with pixel size of 22.5 × 22.5 µm^2^. In order to extract information on the different types of contrast provided by the technique, from three (Zhong *et al.*, 2000 ▸) or five (Rigon *et al.*, 2007 ▸) to several images (Pagot *et al.*, 2003 ▸) taken at different angular positions on the analyzer crystal rocking curve are needed. Therefore, three images of a polypropyl­ene tube (external and internal diameters of 6.0 and 3.8 mm, respectively) taken at different angular positions on the forward-diffracted o-beam rocking curve [positions (i), (ii) and (iii) on the red curve in Fig. 5 ▸(*b*)] were acquired [Figs. 5 ▸(*c*)–5(*e*)]. The results clearly present different contrasts at different angular positions on the forward-diffracted o-beam rocking curve of the Bragg-case analyzer crystal, as expected. This is proof that the forward-diffracted o-beam, in the Bragg case, can be used for ABI in a geometry very similar to the conventional attenuation radiography setup (the detector surface is set after the sample and normal to the X-ray beam), however with differential phase contrast effects (owing to the analyzer crystal which is set between the sample and detector) and with a closed-loop feedback intensity system (at the diffracted grazing emergence h-beam) in order to keep the analyzer crystal angular position fixed.

If one looks at the image cross sections there are no sharp borders, as expected from standard ABis. Such behavior can be due to angular instabilities, or even the stresses in the crystal bulk which can produce some curvatures in the wavefields (mirage effect) inside the crystal (Authier, 2001 ▸; Yan & Noyan, 2006 ▸; Hönnicke & Cusatis, 2007 ▸; Fukamachi *et al.*, 2009 ▸). To further investigate this, forward-diffracted o-beam double-crystal topography on the Bragg-case analyzer crystal was carried out.

## 4. Forward-diffracted o-beam double-crystal topography

**Figure 6:**
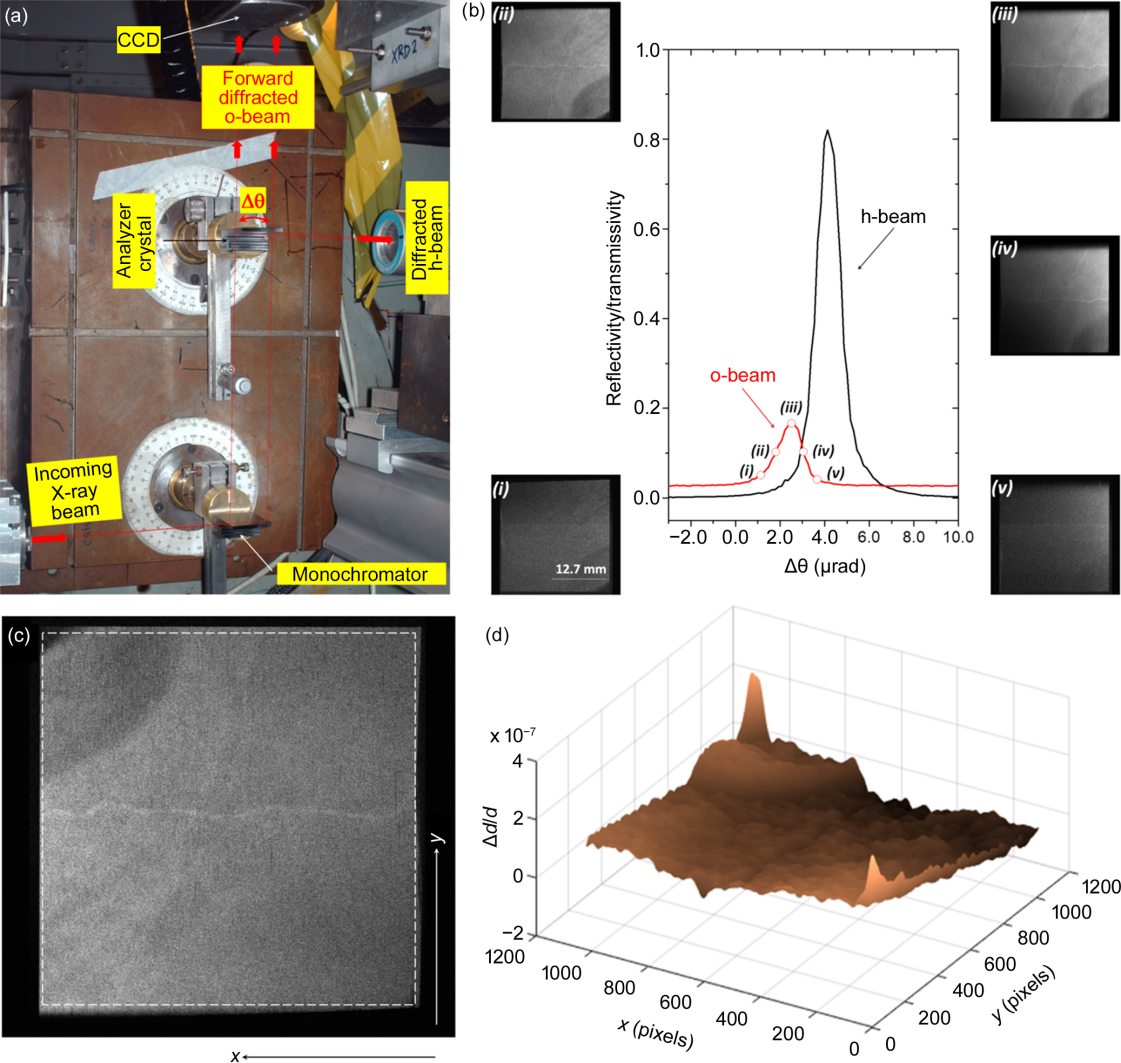
(*a*) Forward-diffracted o-beam double-crystal topography setup mounted in the double-axis diffractometer. The forward-diffracted o-beam is imaged by a CCD detector, while the diffracted h-beam is monitored by a scintillation detector. (*b*) Forward-diffracted o-beam double-crystal topography images (i)–(v) taken at different angular positions on the 3 mm-thick asymmetric Si analyzer crystal with 444 reflection. (*c*) Zoomed-in forward-diffracted o-beam double-crystal topography taken at the maximum positive slope position (ii) on the on the 3 mm-thick asymmetric Si analyzer crystal with 444 reflection and (*d*) the corresponding interplanar variation distance (Δ*d*/*d*) map.

In order to evaluate the stresses in the crystal bulk caused by the crystal’s own weight, an X-ray topography setup was mounted. The setup is basically the same setup presented in the previous section, however without the sample between the first crystal and the analyzer crystal [Fig. 6 ▸(*a*)]. Then, several different X-ray topography images, taken at different angular positions on the forward-diffracted o-beam rocking curve of the Bragg-case analyzer crystal, were collected [Fig. 6 ▸(*b*)]. From the topography images there are clearly stressed areas, seen as different contrast in the images, which should be presented as homogeneous. To quantify the level of stress, an interplanar variation distance (Δ*d*/*d*) map (Lübbert *et al.*, 2000 ▸; Lübbert *et al.*, 2005 ▸) was built based on the topography [Fig. 6 ▸(*c*)] taken at the maximum positive slope position on the forward-diffracted o-beam rocking curve of the Bragg-case analyzer crystal [position (ii) in Fig. 6 ▸(*b*)]. For building the Δ*d*/*d* map, the Si 444 forward back-diffraction o-beam rocking-curve was considered as a Gaussian profile with the same full width at half-maximum (FWHM) as the measured rocking curve [Fig. 4 ▸(*b*), FWHM = 1.18 µrad]. Then, one can employ the following expression to determine the angular deviation (Δθ) of the different pixels on the image:
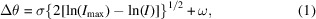
where σ is the width of the Gaussian profile, *I* is the intensity at the different image pixels, *I*_max_ is the maximum intensity of the image and ω is the angular position on the rocking curve where the image was acquired. From the different values for Δθ one can determine Δ*d*/*d*:
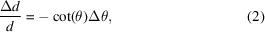
where θ is the diffraction angle. The resulting Δ*d*/*d* map is shown in Fig. 6 ▸(*d*), where variations on the order of 10^−7^ were found. The detected Δ*d*/*d*, caused mainly by the stresses in the crystal bulk, due to the crystal’s own weight could also be detected by the forward back-diffraction o-beam rocking-curve. As previously mentioned, the experimental rocking curve width is FWHM = 1.18 µrad [Fig. 4 ▸(*b*)] while the theoretical rocking curve FWHM = 0.60 µrad [Fig. 1 ▸(*b*)], *i.e.*Δ*d*/*d* ≃ 5.7.10^−7^ in accordance with the Δ*d*/*d* map results. The Δ*d*/*d* variations on the different areas within the analyzer crystal change the intensity locally and, as a consequence, the angular position. This produces local intensity changes in the ABis which can explain the absence of expected sharp borders [Figs. 5 ▸(*c*)–5(*e*)]. Local intensity changes are not detected in the ABis if the crystals have homogeneous Δ*d*/*d*.

## 5. Conclusions and perspectives

A proof of principle ABI experiment based on the forward-diffracted o-beam in a few-millimetre-thick Bragg-case asymmetrically cut analyzer crystal has been implemented and tested in a geometry very similar to that used in conventional radiography. The high angular stability requirements were overcome using specially designed crystals and a closed-loop feedback intensity system, monitoring the intensity of the diffracted h-beam. ABis taken at different angular positions on the forward-diffracted o-beam rocking curve show different contrasts, as expected. However, the ABis did not show sharp borders. This was closely investigated by X-ray topography and Δ*d*/*d* mapping and was attributed to the stresses due to the analyzer crystal’s own weight in the bulk. Further investigation on the crystal design using finite element analysis coupled with the dynamical theory of X-ray diffraction (Cusatis *et al.*, 2022 ▸) is envisaged in order to minimize the stresses due to the analyzer crystal’s own weight.
